# Effect of multiple firings on optical and mechanical properties of Virgilite-containing lithium disilicate glass-ceramic of varying thickness

**DOI:** 10.1007/s00784-024-05746-8

**Published:** 2024-06-13

**Authors:** Amr Rizk, Ahmed Abdou, Reem Ashraf, Sarah Omar

**Affiliations:** 1 Department of Prosthetic Dentistry, Fixed Prosthodontics Division, Faculty of Dentistry, King Salman International University, South Sinai, Egypt; 2 Department of Restorative Dentistry, Faculty of Dentistry, Universiti Malaya, Kuala Lumpur, Malaysia; 3 Department of Prosthetic Dentistry, Dental Biomaterials Division, Faculty of Dentistry, King Salman International University, South Sinai, Egypt; 4 Department of Prosthodontics, Faculty of Oral and Dental Medicine, Badr University in Cairo, Cairo, Egypt

**Keywords:** Lithium disilicate, Color stability, CIEDE2000, Flexure strength, Energy dispersive X-ray

## Abstract

**Objectives:**

To investigate the effect of multiple firings on color, translucency, and biaxial flexure strength of Virgilite-containing (Li_0.5_Al_0.5_Si_2.5_O_6_) lithium disilicate glass ceramics of varying thickness.

**Materials and methods:**

Sixty discs were prepared from Virgilite-containing lithium disilicate blocks. Discs were divided according to thickness (*n* = 30) into T_0.5_ (0.5 mm) and T_1.0_ (1.0 mm). Each thickness was divided according to the number of firing cycles (*n* = 10); F_1_ (Control group): 1 firing cycle; F_3_: 3 firing cycles, and F_5_: 5 firing cycles. The discs were tested for color change (ΔE_00_) and translucency (TP_00_) using a spectrophotometer. Then, all samples were subjected to biaxial flexure strength testing using a universal testing machine. Data were collected and statistically analyzed (α = 0.5). For chemical analysis, six additional T_0.5_ discs (2 for each firing cycle) were prepared; for each firing cycle one disc was subjected to X-ray diffraction analysis (XRD) and another disc was subjected to Energy dispersive X-ray spectroscopy (EDX) and Scanning electron microscope (SEM).

**Results:**

Repeated firing significantly reduced the translucency of F_3_ and F_5_ compared to F_1_ in T_0.5_ (*p* < 0.001), while for T_1.0_ only F_5_ showed a significant decrease in TP_00_ (*p* < 0.001). For ΔE_00_, a significant increase was recorded with repeated firings (*p* < 0.05) while a significant decrease resulted in the biaxial flexure strength regardless of thickness.

**Conclusions:**

Repeated firings had a negative effect on both the optical and mechanical properties of the Virgilite-containing lithium disilicate glass ceramics.

**Clinical relevance:**

Repeated firings should be avoided with Virgilite-containing lithium disilicate ceramics to decrease fracture liability and preserve restoration esthetics.

**Supplementary Information:**

The online version contains supplementary material available at 10.1007/s00784-024-05746-8.

## Introduction

Over the last few decades, dental ceramics have been rapidly evolving, in both structural properties and manufacturing techniques. Among these developments is the introduction of glass ceramics, which combines both high esthetics and exceptional mechanical properties [1]. The major crystalline phase of lithium disilicate (LDS) is Li_2_Si_2_O_5_, with an interlocking elongated crystals microstructure, demonstrating superior esthetics combined with high mechanical strength allowing for the fabrication of a wide range of different fixed prosthetic restorations than previously introduced ceramic materials [2–5].

With the evolvement of CAD/CAM (computer-aided design and computer-aided manufacturing) technology, lithium disilicate blocks were introduced to be milled as a chair-side restorative option which offered reduced laboratory steps, rendering monolithic restorations that do not require veneering by a more esthetic material [6]. This evolvement in digital dentistry in both technology and materials led to a paradigm shift for both clinical practitioners and dental technicians as chair-side indirect restoration became a feasible solution in everyday dental practice [7].

Several attempts to exalt the strength and esthetics of conventional lithium disilicate were endeavored by altering its microstructure, composition, and/or particle size. Recently, a new CAD/CAM block known as advanced lithium disilicate (ALD) has been introduced in dental practice. This ceramic material is characterized by quick firing time accompanied by enhanced strength and esthetics through incorporating Virgilite (Li_0.5_Al_0.5_Si_2.5_O_6_) and zirconia within a lithium disilicate glass-ceramic matrix [8, 9]. Virgilite is a needle-like crystal (0.5 μm length) [10] which gives the same level of strength as rebar buried in cement. These crystals are activated by matrix firing which boosts the material’s density and strength by inhibiting crack propagation and preventing restoration fracture while preserving the great esthetic properties of ceramic restoration which mimics natural tooth appearance [9].

The dental ceramic microstructure is the paramount factor in defining their translucency. Alteration could be achieved by modifying the crystals by changing their volume, size, and/or density. To improve the translucency of glass ceramics, a small-grained microstructure is desirable [11]. Crystal formation in ceramics depends on the presence of nucleating agents that will tailor the crystal growth and finally determine the shape, size, and composition of each crystal [12]. Moreover, ceramic translucency can be altered by adjusting the glass composition and microstructure. Tailoring pigments, additives, and heat treatment controls both translucency and strength [13, 14]. Furthermore, the mechanical properties of ceramics are influenced by the crystal size [15], crystalline contents, and the irregularity of particles [16].

During the laboratory process, restorations might require adjustments either to add or remove a part of the restoration to modify its form, shape, and/or color through add-on, layering and staining or even grinding, finishing, and glazing [17]. In addition, during the clinical scenario, such restoration might require adjustments during the try-in process. Those modifications may require an extra firing step to achieve an esthetically pleasing restoration. Dental ceramics are affected by prolonged/repeated heat application which might alter the optical and mechanical properties of the ceramic material [17, 18].

Therefore, this study aims to evaluate the effect of multiple firings on color, translucency, and biaxial flexural strength of Virgilite-based advanced lithium disilicate of different thicknesses. The first null hypothesis of this study was that the optical properties of the advanced lithium disilicate would not be affected by multiple firing for varying thicknesses. The second null hypothesis was that the biaxial flexural strength would not be affected by repeated firing cycles.

## Materials and methods

### Sample size and disc grouping

Based on data extracted from Abdel Sadek et al [19], a minimum sample size of 24 samples (*n* = 8 in each group) is enough with effect size d = 0.8868241 and results in 95% power when the significant level is 0.05. The sample size was increased to 10 samples in each group for statistical analysis reliability.

**Fig. 1:**
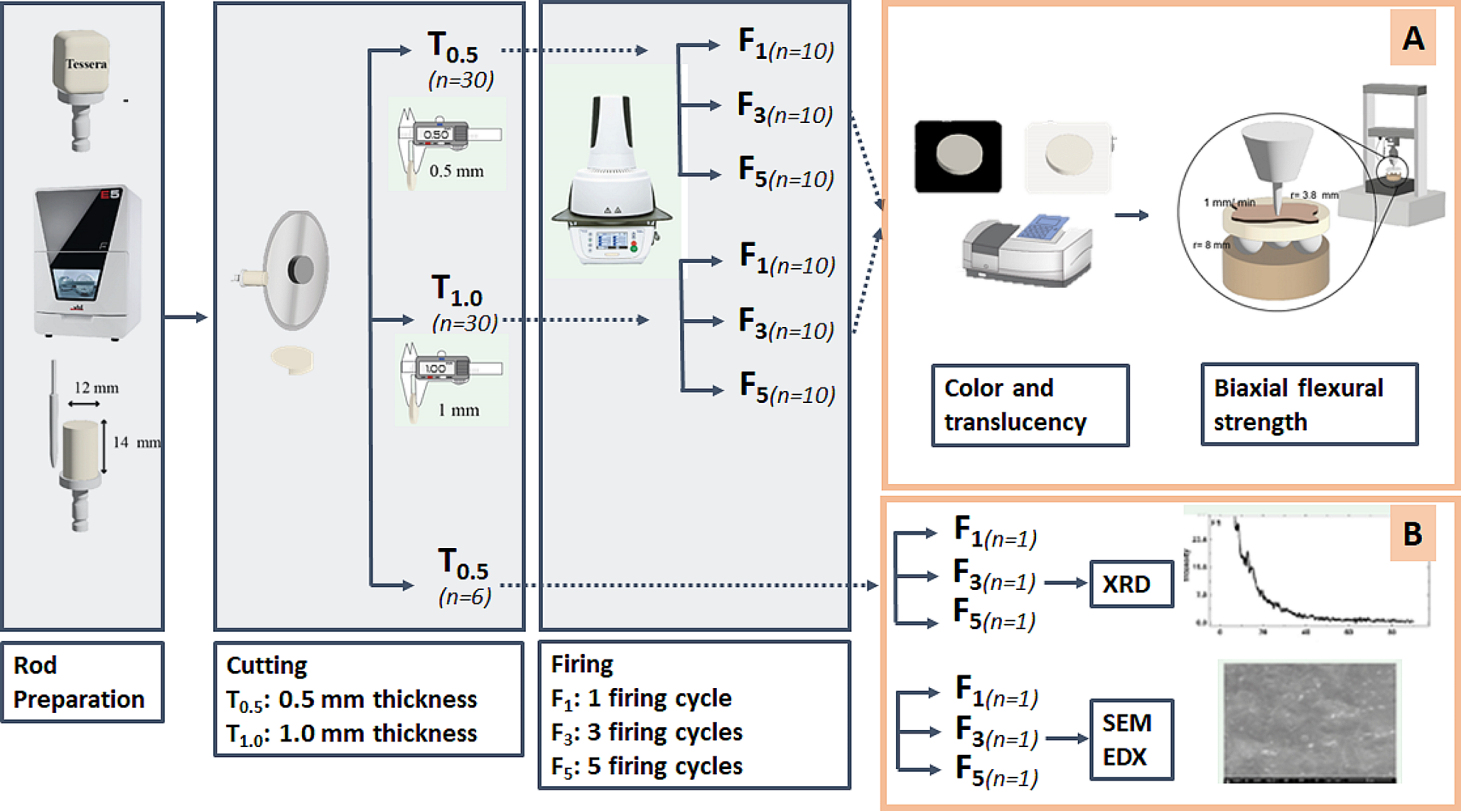
A schematic diagram for the grouping and experimental design. **A**. Samples were tested for color parameters, followed by biaxial flexure strength. **B**. Chemical analysis for each group

A total of 66 Virgilite-containing lithium disilicate discs (12 mm diameter), (CEREC Tessera, Dentsply/Sirona, Germany) were constructed, and the discs were allocated into two groups according to thickness (*n* = 30) into T_0.5_ (0.5 mm) and T_1.0_ (1.0 mm). For both optical properties and biaxial flexure strength measurements, 60 discs were used and divided according to the number of firing cycles (*n* = 10, each): F_1_; control group [subjected to 1 glaze firing], F_3_; subjected to 1 glaze firing followed by 1 add-on firing and then 1 glaze firing, and F_5_; subjected to 5 firing cycles [first and last cycles are glaze firings with intervening 3 add-on firings]. For chemical analysis, 6 additional discs of T_0.5_ were used. Specimen grouping and distribution are summarized in Fig. 1.

### Discs preparation

A cylinder of 14 mm in length and 12 mm in diameter was designed as an STL file, using design software (3D Builder, Microsoft Corporation, USA). The STL file was imported to a CAD design software (Exocad GmbH, Germany). A CEREC Tessera CAD/CAM block (A2 HT) was mounted into a 5-axis milling machine (VHF, VHF International, Ammerbuch, Germany) and the milling process was initiated, utilizing a wet milling protocol.

The milled cylinder was cut using a linear precision saw (IsoMet 4000, BUEHLER LTD, USA) producing a disc of 0.5 mm and 1.0 mm. The sawing was done under copious cooling to prevent the burning of the material or the induction of cracks. The dimensions of the discs were verified using a digital caliper (Mitutoyo, Kanagawa, Japan), and any defective discs were discarded.

### Discs firing

The samples were fired using Programat EP 5000 Furnace (Ivoclar Vivadent, Germany) following the manufacturer’s recommendation. (see appendix)

### Optical properties measurement

The optical properties (color and translucency) were measured for each group using a reflective spectrophotometer (UV- Shimadzu 3101 PC, Shimadzu, Japan) against white (CIE L* = 88.81, a* = −4.98, b* = 6.09) and black (CIE L* = 7.61, a* = 0.45, b* = 2.42) backgrounds relative to the CIE standard illuminant D65. The CIEDE2000 (ΔE_00_) color difference of each specimen was calculated using the following formula: [20]

$$\Delta {E_{00}} = {\left[ \begin{gathered}{\left( {\frac{{\Delta L\prime }}{{{K_L}{S_L}}}} \right)^2} + {\left( {\frac{{\Delta C\prime }}{{{K_C}{S_C}}}} \right)^2} + {\left( {\frac{{\Delta H\prime }}{{{K_H}{S_H}}}} \right)^2} \\ + {R_T}\left( {\frac{{\Delta C\prime }}{{{K_C}{S_C}}}} \right)\left( {\frac{{\Delta H\prime }}{{{K_H}{S_H}}}} \right) \\ \end{gathered} \right]^{\frac{1}{2}}}$$

For a pair of samples in CIEDE2000, *ΔL’*, *ΔC’*, and *ΔH’* are the differences in lightness, chroma, and hue.

*R*_*T*_ is a rotation function that accounts for the interaction between chroma and hue differences in the blue region.

Weighting functions, *S*_*L*_, *S*_*C*_, *S*_*H*_, adjust the total color difference for variation in the location of the color difference pair in *L’, a’, b’* coordinates and the parametric factors *K*_*L*_, *K*_*C*_, *K*_*H*_ are correction, terms for experimental conditions.

The translucency parameter (TP_00_) values were attained by calculating the difference in the color of the samples that were measured against black and white backgrounds using the following formula: [20]

$$T{P_{00}} = {\left[ \begin{gathered}{\left( {\frac{{L{\prime _B} - L{\prime _W}}}{{{K_L}{S_L}}}} \right)^2} + {\left( {\frac{{C{\prime _B} - C{\prime _W}}}{{{K_C}{S_C}}}} \right)^2} + {\left( {\frac{{H{\prime _B} - H{\prime _W}}}{{{K_H}{S_H}}}} \right)^2} \\ + {R_T}\left( {\frac{{C{\prime _B} - C{\prime _W}}}{{{K_C}{S_C}}}} \right)\left( {\frac{{H{\prime _B} - H{\prime _W}}}{{{K_H}{S_H}}}} \right) \\ \end{gathered} \right]^{\frac{1}{2}}}$$

TP_00_ = translucency parameter.

The subscripts “B” and “W” refer to the color coordinates against black and white backgrounds, respectively.

### Biaxial flexural strength

The same samples used for translucency evaluation were used (*n* = 10, in each group) for biaxial flexural strength testing. Testing was conducted using a ball-on ring fixture at a crosshead speed of 1 mm/min with a computer-controlled materials testing machine (Model 3345; Instron Industrial Products, Norwood, MA, USA) with a loadcell of 5 kN. Also, data was listed using computer software (Instron® Bluehill Lite Software). Discs were placed on 8 mm diameter circular knife-edge support and loaded centrally with a spherical indenter of 3.8 mm diameter. A thin sheet of rubber was placed between each sample and the load applicator tip to facilitate a uniform load distribution. The biaxial flexure strength was calculated according to the following equation:

$$\sigma =P/\left({h}^{2} \right\{(1+\nu )[0.485\times in(a/h)+0.52]+0.48\left\}\right)$$

where σ; the biaxial flexure strength (MPa), P; the measured load at fracture (N), a; the radius of the circular knife-edge support (mm), h; the specimen thickness and *v* the Poisson’s ratio for the material.

### X-ray diffraction analysis (XRD)

Another 3 samples (0.5 mm) representative of each firing cycles protocol were constructed as previously mentioned. Samples were subjected to XRD analysis using an X-ray diffractometer (LabX XRD-6000, Shimadzu, USA). XRD patterns were obtained in the range of 2θ from 4° to 90° at room temperature. Cu Kα was used as a radiation source of wavelength λ = 0.15408 nm and the operating conditions included scanning rate of 8°/min., and the operation voltage and current were 40 kV and 30 mA, respectively.

### Scanning electron microscope analysis (SEM) and Energy dispersive X-ray analysis (EDX)

Three additional samples, each measuring 0.5 mm in thickness, were made specifically for the SEM/EDX evaluation. The surface imaging was performed under a scanning electron microscope (ZEISS-EVO 15, ZEISS, UK). Images were captured at 2,500x and 10,000x magnifications. EDX analysis was carried out using an X-ray detector attached to a scanning electron microscope. Each sample was subjected to an energetic electron beam (25 keV) where the resulting characteristic X-rays emitted from the sample surface were utilized in determining the constituent elements quantitatively.

### Statistical analysis

Data showed normal distribution, so One-Way ANOVA was used to compare between the number of repeated cycles within each thickness. Also, an independent t-test was used to compare different thicknesses within each firing cycle group for TP_00_ and color parameters. Biaxial flexure strength data were analyzed with Weibull analysis (R4.1, R: A language and environment for statistical computing. R Foundation for Statistical Computing, Vienna, Austria). Wald estimation was used; moreover, different groups were compared at the Weibull characteristic strength (63.2% probability of failure). The significance level was set at *p* = 0.05.

## Results

### Translucency results

TP_00_ data is represented in Table 1. For T_0.5_, F_3_ and F_5_, a significant decrease in the translucency compared to F_1_ (*p* < 0.001) was observed. T_1.0_, F_5,_ showed significantly lower translucency compared to F_1_ and F_3_. In general, T_1.0_ showed lower translucency compared to T_0.5_ irrespective of the number of firing cycles (*p* < 0.05).

**Table 1:** Mean and standard deviation of the translucency (TP_00_) for different thicknesses and firing cycles

	F_1_	F_3_	F_5_	*p*-value
T_0.5_	19.18^a^ ± 2.33	14.01^b^ ± 2.22	13.76^b^ ± 1.93	< 0.001
T_1.0_	11.90^a^ ± 1.92	9.80^a^ ± 1.64	7.12^b^ ± 1.21	< 0.001
*p*-value	< 0.001	< 0.001	< 0.001	

Different letters within each row indicate a significant difference (Tukey HSD, *p* < 0.05). Comparison between thickness for each firing cycle is represented by the column p-value

### Color results

For ΔL, T_0.5_ showed a significantly higher ΔL value compared to T_1.0_ after F_3_ (*p* = 0.007); however, the increase in ΔL was insignificant after F_5_ (*p* = 0.06). Yet, it was at the significance level borderline. For both thicknesses, ΔL showed a significantly higher value after F_5_ compared to F_3_.

For ΔC, T_0.5_ showed significantly lower values compared to T_1.0_ after both F_3_ and F_5_ (*p* = 0.002 and < 0.001). For T_0.5_, ΔC showed a significantly higher value after F_5_ compared to F_3_ (*p* = 0.016). Similarly, the change in ΔC was significant for T_1.0_ (*p* = 0.004).

For ΔH, the change in thickness did not result in a significant change in ΔH values after F_3_ (*p* = 0.835) while for F_5_, significantly lower ΔH values resulted for T_1.0_ compared to T_0.5_ (*p* = 0.016). For T_0.5_, a significantly higher value after F_5_ compared to F_3_ (*p* = 0.002) while an insignificant difference resulted for T_1.0_ (*p* = 0.153).

For ΔE_00_, firing cycles resulted in a significant increase in ΔE_00_ values (*p* < 0.05). For F_3_, insignificant differences resulted between T_1.0_ and T_0.5_ at *p* = 0.054. however, the *p*-value is borderline. For F_5_, insignificant differences resulted between T_1.0_ and T_0.5_ at *p* = 0.117 Color parameters are presented in Table 2. The change in color parameters between F3 and F5 can be seen in the appendix.

**Table 2:** Mean and standard deviation for different color parameters

		F_1_ Vs. F_3_	F_1_ Vs. F_5_	*p*-value
*ΔL*	T_0.5_	0.24 ± 0.05	0.79 ± 0.06	< 0.001
	T_1.0_	0.46 ± 0.06	1.17 ± 0.25	0.008
*p*-value		0.007	0.06	
*ΔC*	T_0.5_	0.36 ± 0.17	0.82 ± 0.10	0.016
	T_1.0_	1.23 ± 0.11	2.18 ± 0.25	0.004
*p*-value		0.002	< 0.001	
*ΔH*	T_0.5_	1.24 ± 0.10	2.06 ± 0.16	0.002
	T_1.0_	1.21 ± 0.15	1.47 ± 0.20	0.153
*p*-value		0.835	0.016	
*ΔE*_*00*_	T_0.5_	1.19 ± 0.12	2.1 ± 0.16	0.002
	T_1.0_	1.50 ± 0.16	2.3 ± 0.06	0.001
*p*-value		0.054	0.117	

Comparison between both thicknesses for each parameter indicated by the p-value (α = 0.05)

### Biaxial flexure strength

**Fig. 2:**
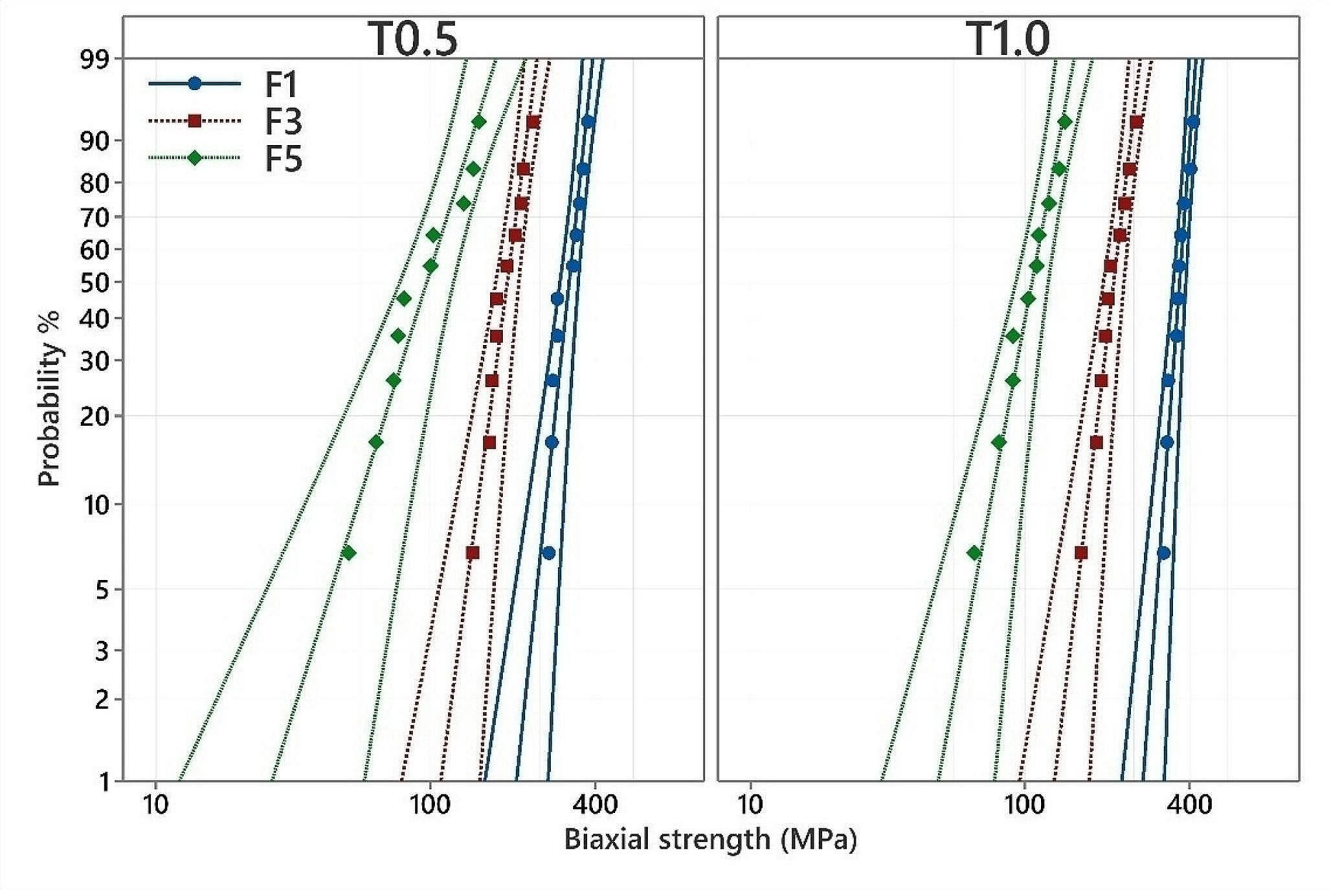
The Weibull probability plot of the biaxial flexure strength (MPa) of the tested groups

Results of biaxial flexure strength are presented in Table 3; Fig. 2. The highest Weibull characteristic strength resulted for T_1.0_F_1_ followed by T_0.5_F_1_ with a significant difference between each other. F_3_ showed a significant difference with F_1_ and F_5_ for both thicknesses. F_5_ showed the lowest Weibull characteristic strength for both thicknesses compared to all other groups. In general, increasing the firing cycles resulted in decreasing the Weibull characteristic strength. After F_3_ and F_5_, specimen thickness revealed no significant effects on the Weibull characteristic strength, whereas T_0.5_ showed a significantly lower Weibull characteristic strength compared to T_1.0_.

**Table 3:** The results of Weibull analysis of biaxial flexure strength

Cycles	Thickness	Mean ± SD	α [95% CI]	β [95% CI]	P10 [95% CI]
F_1_	T_0.5_	316.86 ± 38.97	333.6 [311.5 to 357.3]^c^	9.6 [6.4 to 18.7]	263.7 [226.5 to 307]
F_1_	T_1.0_	363.56 ± 29.94	376.9 [359.3 to 395.3] ^d^	13.8 [9.4 to 26.1]	320.1 [288.8 to 354.9]
F_3_	T_0.5_	188.85 ± 29.04	200.9 [184.2 to 219.1] ^b^	7.6 [5.1 to 14.5]	149.3 [123.5 to 180.4]
F_3_	T_1.0_	208.73 ± 28.82	220.8 [204.4 to 238.5] ^b^	8.5 [5.7 to 16.3]	169.4 [143.1 to 200.5]
F_5_	T_0.5_	97.35 ± 34.75	108.9 [89 to 133.3] ^a^	3.3 [2.2 to 6.3]	54.5 [34.9 to 85.1]
F_5_	T_1.0_	104.95 ± 23.71	114 [100.9 to 128.8] ^a^	5.4 [3.6 to 10.5]	74.9 [57.1 to 98.2]

Different superscript letters within the α column are statistically significant differences based on 95% confidence interval (CI). α: characteristic strength or scale of a Weibull parameter. β: the shape, slope, and modulus of a Weibull parameter. P10: estimation at 10% probability of failure

### XRD and EDX

**Fig. 3:**
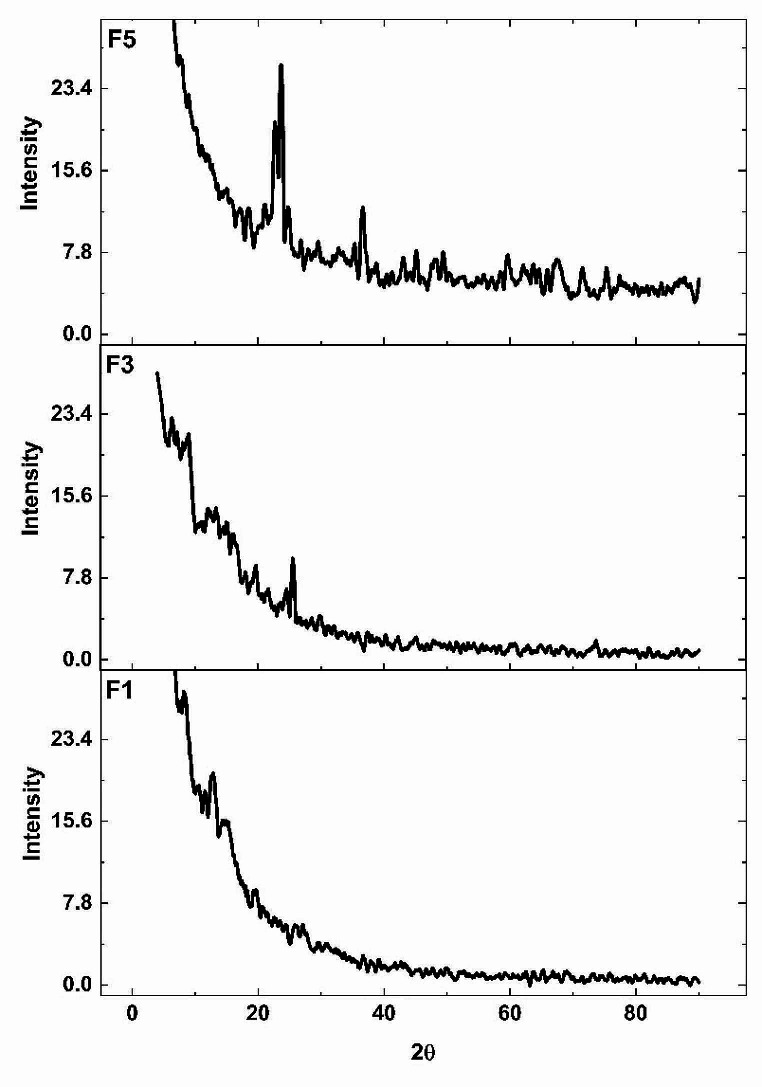
XRD analysis

A higher peak intensity for lithium disilicate crystals (Li_2_Si_2_O_5_) at 24–26 2θ and 38 2θ resulted in F_5_ compared to F_1_. Additionally, peaks for Li_3_PO_4_ showed at 22–24 and 34–37 2θ for both F_3_ and F_5_. The peaks of Virgilite crystals are overleaped with quartz at 27, 41, and 45 2θ as both have a similar crystal structure [21]. For all the peaks detected, the intensity was higher for F_5_ compared to F_1_. For the EDX analysis, Na showed a continuous decrease in the wt %; meanwhile, Al has shown a continuous increase in wt % with repeated firing cycles. EDX analysis is presented in Table 4 and XRD in Fig. 3.

**Table 4:** EDX analysis of different groups

	Li	C	O	Na	Mg	Al	Si	*P*	K	Zn
F_1_	0.94	7.97	66.82	1.64	0.62	3.37	16.48	0	1.29	0.06
F_3_	0.76	6.49	68.14	1.19	0.66	4.15	16.27	0.03	1.31	0.05
F_5_	0.64	2.78	60.52	0.02	0.92	6.21	25.49	0	1.46	0.08

### Scanning electron microscope (SEM)

**Fig. 4:**
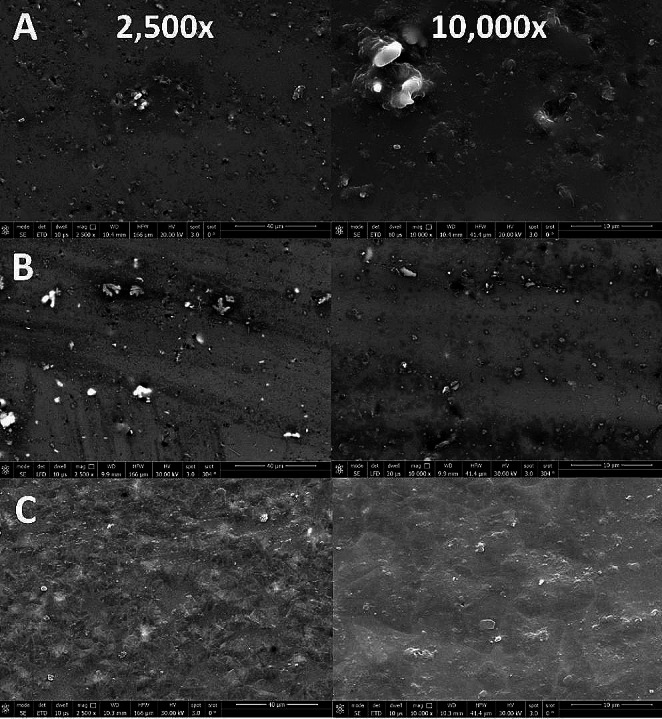
Representative scanning electron microscope images for tested groups at 2,500x (left-hand side) and 10,000x (right-hand side): A: F_1_ (1 firing cycle); B: F_3_ (3 firing cycles); and C: F_5_ (5 firing cycles)

The surface morphology of Advanced Lithium Disilicate sections is shown in Fig. 4. Figure 4A, represents 1 firing cycle where the surface shows a surface porosity with the presence of the opaque crystalline Virgilite component of the ALD.

Meanwhile, in Fig. 4B, after 3 firing cycles, the surface is relatively dense and intact compared with Fig. 4A. A particular feature was observed on the surface where the glassy matrix appeared to form an interlocking pattern in some sites with a reduction in porosities.

Regarding Fig. 4C, the surface has shown that the 5 firing cycles of ALD have led to large crystals size and the interlocking pattern eventually disappeared and is now replaced by a dense nonporous surface with dispersed Virgilite crystals over the crystalline matrix.

## Discussion

The first null hypothesis of the study was rejected as the repeated firings significantly changed the translucency of T_0.5_F_3,_ T_0.5_F_5,_ and T_1.0_F_5_ with no significant change on T_1.0_F_3_. The second null hypothesis of the study was rejected as the biaxial flexure strength of the ceramic discs decreased on repeating the firing cycles, which was more evident in T_0.5_ groups. This could be attributed to the changes in the crystalline structure and grain size of the material that is affected by temperature or duration of exposure to firing temperature as a result of repeated firings.

The chemical composition of ALD holds natural mineral Virgilite crystals, which are claimed to have a synergistic effect with the lithium disilicate altogether with zirconia oxide embedded in a glassy matrix. The contained crystals are small in size (lithium disilicate crystals of 0.5 μm and Virgilite crystals of 0.2–0.3 μm) in comparison to conventional LDS (lithium disilicate crystals > 1 μm); this will have a major impact on the material’s enhanced optical and mechanical properties [22, 23].

In general, repeated firing increases the material exposure time to high temperature and will lead to mobility of molecules, lowering the glass viscosity, and facilitating dislocation, and thereby forming crystal aggregates. This may cause further crystallization and nucleation of the smaller crystals with zirconia acting as nucleating agents, leading to the formation of more grain boundaries that might lead to diffuse transmission of light where increasing the grain size is favorable to encountering a less-powerful light beam at the grain boundaries resulting in diffuse scattering of light [11]. Additionally, repeated firing causes more compact interlocking of microstructures of Li_2_Si_2_O_5_ [24]. Moreover, a densely crystalline structure can develop surface damage at high temperatures [25]. This was confirmed by the compact crystalline structure in Fig. 4B, C. Also, EDX depicted an increase in Al wt % with repeated firing that may be attributed to decreased translucency [26].

The reduction in translucency in this study as a result of repeated firing was in agreement with previously conducted studies [19, 27] whose findings were credited to the alteration in crystal size and/or orientation. Also, another study reported an initial increase in translucency with an increased number of firings followed by a decrease in the translucency with increasing the number of sintering cycles. This was justified by the chemical reaction that caused transformation of the glass phase into crystalline phase and the change of crystals arrangement, leading to more light reflection and refraction with increasing the number of sintering cycles [25, 28]. The translucency of 0.5 mm thick samples was significantly affected by 3 and 5 firing cycles, while for 1.0 mm only the change in translucency was significant after 5 firing cycles. This might be attributed to the fact that thinner sections allow for more light to pass through discs, so even the minor microstructure changes resulting from multiple firing leads to more light scattering and may have a profound effect on the thin ceramic sections [29].

This finding was coherent with another study [30], which investigated the effect of multiple firings (3 firings only) on glass ceramics with different thickness (0.6 mm and 1.0 mm) and found that the change in translucency was only significant with thinner sections. On the other hand, other studies [31, 32] investigated the effect of multiple firings on the translucency of ceramic materials and they found that multiple firings did not affect translucency parameters, making their findings in disagreement with our study. The reason for the different findings is that both studies used veneering porcelain build-up to framework rather than a monolithic block as the case in the present study.

Regarding the effect of repeated firings on color change (ΔE_00_), it is agreed that a color difference of ΔE_00_ ≥ 1.77 is considered a poor match color difference, which is clinically unacceptable [33]. The results obtained for F_3_ and F_5_ groups had a statistically significant higher mean (ΔE_00_) value in comparison to F_1_ group. The values of F_1_ vs. F_3_ for both thicknesses were considered perceivable but still within acceptability range. As for, F_1_ vs. F_5_ for both thicknesses, samples were considered unacceptable [33]. Our results were in agreement with previously conducted studies [34, 35], which investigated the effect of repeated firings on the color change of glass-based ceramics. They reported that the color instability of metal oxides during firings and alteration in surface colorants and pigment breakdown, crystalline growth, and consequent alteration of light refraction may be attributed to the increased color difference [34, 35].

The biaxial flexure strength results showed decreased values with repeated firings. This may be attributed to the increase in the crystalline content and formation of new phases with particles of various and irregular particle sizes that might induce stress, flaws, and break the interfacial interaction between the matrix and particles [36]. In addition, there exists a possibility of having induced cracking as a result of multiple nucleation sites during crystallization, forming new crystals that are coarse together with the new phases with different sizes of crystals, which makes the crystalline pattern irregular and more prone to cracking [37]. This was confirmed by peaks from the XRD analysis that changed on repeated firings, indicating the formation of new crystalline structure as shown for Lithium disilicate crystals (Li_2_Si_2_O_5_) at 24–26 2θ and 38 2θ and Virgilite crystals at 27, 41, and 45 2θ [21] crystals at 24–26 2θ for 5 cycles compared to 1 cycle. Moreover, Na showed a continuous decrease in the wt % which is an indication of its consumption in the formation of sodium silicate crystals and at higher sodium content sodium disilicate (Na_2_Si_2_O_5_) crystals [38]. The microstructural changes caused by repeated firing were evident in SEM images as the grain size became larger and more condensed.

A previous study [19] investigated the effect of multiple firings on zirconia-reinforced glass ceramics, both pressed and machined (Vita Amberia, VITA Zahnfabrik, Germany). They found that multiple firings caused a significant decrease in biaxial flexure strength when it was fired up to 5 times. Our findings disagree with another study that assessed the biaxial flexure strength of 4 different ceramic materials (Zirconia, Zirconia-reinforced lithium silicate, conventional lithium disilicate, and leucite-based glass ceramic) as a result of multiple firings on (2 and 4 firing cycles). They found that the biaxial flexure strength was significantly affected only with zirconia yielding no significant effect on glass-based ceramics [39].

To the best of our knowledge, there are reports concerned with the physical and mechanical behavior of the ALD, but none has correlated physical properties and flexural strength to repeated firing till now. This may be attributed to the novelty of the material that should be further investigated.

In the present study, the used samples were not glazed and had a flat disc shape, which does not conform to the actual shape of dental restoration. Those variables, in addition to the variation in surface texture, should be reinvestigated with proper tooth-form restorations to determine the effect of multiple firings on the optical and mechanical properties of Virgilite-containing lithium disilicate. Another limitation is that aging for samples was not achieved, as restorations are subjected to thermal changes inside the oral cavity which might inflict the assessed tested outcomes. Also, for a restoration to be subjected to 5 firing cycles during a clinical scenario might be impractical. Further studies should investigate the effect of repeated firings on different prosthetic restorations that had been veneered and glazed as well as investigating the effect of simulated aging combined with multiple firings on the tested parameters.

## Conclusions

Taking the results and conditions of this study into consideration, the following conclusions can be drawn:

1. Multiple firings decreased the translucency of Advanced Lithium Disilicate, especially in reduced material thickness.
2. Color change was evident with repeated firing. It was considered clinically acceptable after 3 firring cycles but the color change exceeded the appetence threshold after 5 firing cycles regardless of the thickness.
3. Biaxial flexure strength of Virgilite-based glass-ceramic was affected by the number of firing cycles.
4. Clinicians and dental technicians should decrease corrective adjustments and recontouring of Advanced Lithium Disilicate restorations that necessitate additional firings.

### Electronic supplementary material

Below is the link to the electronic supplementary material.

Supplementary Material 1

## Data Availability

The raw data required to reproduce these findings are available upon reasonable request from the corresponding author. The processed data required to reproduce these findings are available upon reasonable request from the corresponding author.
