# Supplementary material for: Effect of multiple firings on optical and mechanical properties of Virgilite-containing lithium disilicate glass-ceramic of varying thickness
Source: Clin Oral Investig. 2024 Jun 13;28(7):370. doi: 10.1007/s00784-024-05746-8 (PMC11176229; doi:10.1007/s00784-024-05746-8)
Supplement: Supplementary file 1 — Supplementary Material 1 [file 784_2024_5746_MOESM1_ESM.docx]

**Table 1**

Firing parameters

|  | **Glaze** | **ADD ON** |
| --- | --- | --- |
| Pre-Heating Temperature | 400 °C | 400 °C |
| Pre-Heating Time | 2 min | 2 min |
| Heat Rate | 55 °C/min | 55 °C/min |
| Final Temperature | 760 °C | 750 °C |
| Vacuum Holding Time | 0 min | 1 min |
| Holding Time without Vacuum | 2 min | 1 min |

*Color change results*

**Table 2**

Mean and standard deviation for different color parameters

|  | | F_1_ Vs. F_3_ | F_1_ Vs. F_5_ | F_3_ Vs. F_5_ | *p*-value |
| --- | --- | --- | --- | --- | --- |
| ΔL | T_0.5_ | 0.24^c^±0.05 | 0.79^a^±0.06 | 0.55^b^±0.01 | <0.001 |
|  | T_1.0_ | 0.46^b^±0.06 | 1.17^a^±0.25 | 0.71^ab^±0.27 | 0.017 |
| *p*-value | | 0.007 | 0.06 | 0.348 |  |
| ΔC | T_0.5_ | 0.36^b^±0.17 | 0.82^a^±0.1 | 0.47^b^±0.08 | 0.009 |
|  | T_1.0_ | 1.23^b^±0.11 | 2.18^a^±0.25 | 0.95^b^±0.31 | 0.002 |
| *p*-value | | 0.002 | <0.001 | 0.061 |  |
| ΔH | T_0.5_ | 1.24^a^±0.1 | 2.06^c^±0.16 | 0.81^b^±0.09 | <0.001 |
|  | T_1.0_ | 1.21^a^±0.15 | 1.47^a^±0.2 | 0.23^b^±0.19 | <0.001 |
| *p*-value | | 0.835 | 0.016 | 0.009 |  |
| ΔE_00_ | T_0.5_ | 1.19^b^±0.12 | 2.1^a^±0.16 | 0.95^b^±0.05 | <0.001 |
|  | T_1.0_ | 1.5^b^±0.16 | 2.3^a^±0.06 | 0.92^c^±0.25 | <0.001 |
| *p*-value | | 0.054 | 0.117 | 0.863 |  |

Different letter within each row indicates significant differences (Tukey’s adjusted *p*-value).
